# Animal models of oral infectious diseases

**DOI:** 10.3389/froh.2025.1571492

**Published:** 2025-04-17

**Authors:** Jing Li, Ya-Nan Zhao, Yi-Hui Wang, Yao Chen, Jia-Long Hou, Da-Yuan Wang, Linqi Shi, Jing Shen

**Affiliations:** ^1^ Department of International VIP Dental Clinic, Tianjin Stomatological Hospital, School of Medicine, Nankai University, Tianjin, China; ^2^ Tianjin Key Laboratory of Oral and Maxillofacial Function Reconstruction, Tianjin, China; ^3^ Department of Operative Dentistry and Endodontics, Tianjin Stomatological Hospital, School of Medicine, Nankai University, Tianjin, China; ^4^ State Key Laboratory of Medicinal Chemical Biology, Key Laboratory of Functional Polymer Materials, Ministry of Education, Institute of Polymer Chemistry, College of Chemistry, Nankai University, Tianjin, China

**Keywords:** oral infectious disease, animal model, caries, pulpitis, periodontitis, oral candidiasis

## Abstract

Oral infectious diseases, including caries, pulpitis, periodontitis, and oral candidiasis, are caused by plaque biofilm or dysbiosis. These conditions affect over two billion people worldwide, imposing a significant burden on healthcare systems and economies. Developing suitable animal models is crucial for investigating the underlying mechanisms of these diseases and evaluating potential therapeutic strategies. Currently, most animal models of oral infectious diseases are built via inoculating a single pathogenic bacterium. However, these models often fail to fully replicate the complex disease processes observed in humans. As a result, alternative methods are needed to explore more accurate animal models that better represent the progression of oral infectious diseases. Herein, this mini-review provides a concise overview of strategies for constructing animal models of oral infectious diseases, focusing on four representative conditions: caries, pulpitis, periodontitis, and oral candidiasis. The goal is to offer valuable insights and references for researchers working in the field of animal model development for oral health.

## Introduction

The microbiome in the human oral cavity is complex, consisting of various microorganisms including bacteria, fungi, viruses, archaea and protozoa, approximately 700 species of prokaryotes have been identified in them (1). Homeostasis of the oral microbiome plays a pivotal role in maintaining health. They establish a distinct oral microenvironment regulated through sophisticated signalling systems as well as host and environmental factors. Once the balance of the oral microbial community is disrupted, the predominant pathogens can seize the opportunity to trigger various oral infectious diseases (2), such as pulpitis, caries, periodontitis, and oral candidiasis (3). According to The Systematic Analysis of Global Burden of Oral Conditions, oral infectious diseases are one of the most prevalent infectious diseases globally, imposing a significant economic burden on patients and having a substantial effect on their overall health and quality of life (4). Even worse, the oral microbes can translocate to the rest of the body through the oral-gut axis, exerting profound effects on systemic health and enhancing the possibility of cardiovascular events, neurological disorders, autoimmune diseases, diabetes, and cancer (5–9). Therefore, it's of great necessity to deeply explore the pathogenic mechanisms of oral infectious diseases and verify their therapeutic effects *in vivo*. It will lay a solid foundation for formulation of subsequent prevention and treatment strategies. From an ethical perspective, animal models can be used as alternatives to mimic the complex human biological processes and examine the microbiome changes that occur during the course of a disease, from its initial onset to its progression, under relatively controllable conditions (10). However, as sentient beings, the welfare and rights of animals must be respected. The establishment of the rule of the three Rs-replacement, reduction and refinement needs to be taken seriously.

Oral infectious diseases involve a complex and diverse microbial environment and are closely related to the host immune response, making the construction of an ideal animal model for oral infectious diseases difficult and challenging. Most of the current animal models are constructed by inoculation with major pathogenic bacteria, but the diseases induced by the above methods are different from those that occur naturally in humans. To better stimulate infectious diseases, researchers have employed methods such as inoculating mixed bacterial suspensions, drug stimulation, transplanting human saliva or plaque into the oral cavities of animals, or combining multiple approaches. Animal models are often based on rodents, pigs, dogs, rabbits, monkeys, etc. The dental structures, oral microbiota and immune responses of non-human primates (monkeys) are highly similar to those of humans, enabling them to more realistically simulate human oral infectious diseases. However, primates are highly intelligent and social, due to ethical considerations on animal welfare and legal frameworks for animal research, the use of these large animals is increasingly restricted (11). In contrast, rodents are inexpensive, easy to handle, and can be genetically manipulated. Despite some differences in anatomy and immune system compared to humans, they are still widely used as animal models for the study of oral infectious diseases. This mini-review will provide a succinct summary of the strategies for constructing animal models of oral infectious diseases from four representative aspects: caries, pulpitis, periodontitis and oral candidiasis. This article will provide valuable references and insights for the research on animal models in related fields. It comprehensively reviews the existing construction methods, analyzes their advantages and disadvantages, and explores the emerging trends.

## Caries

**Figure 1:**
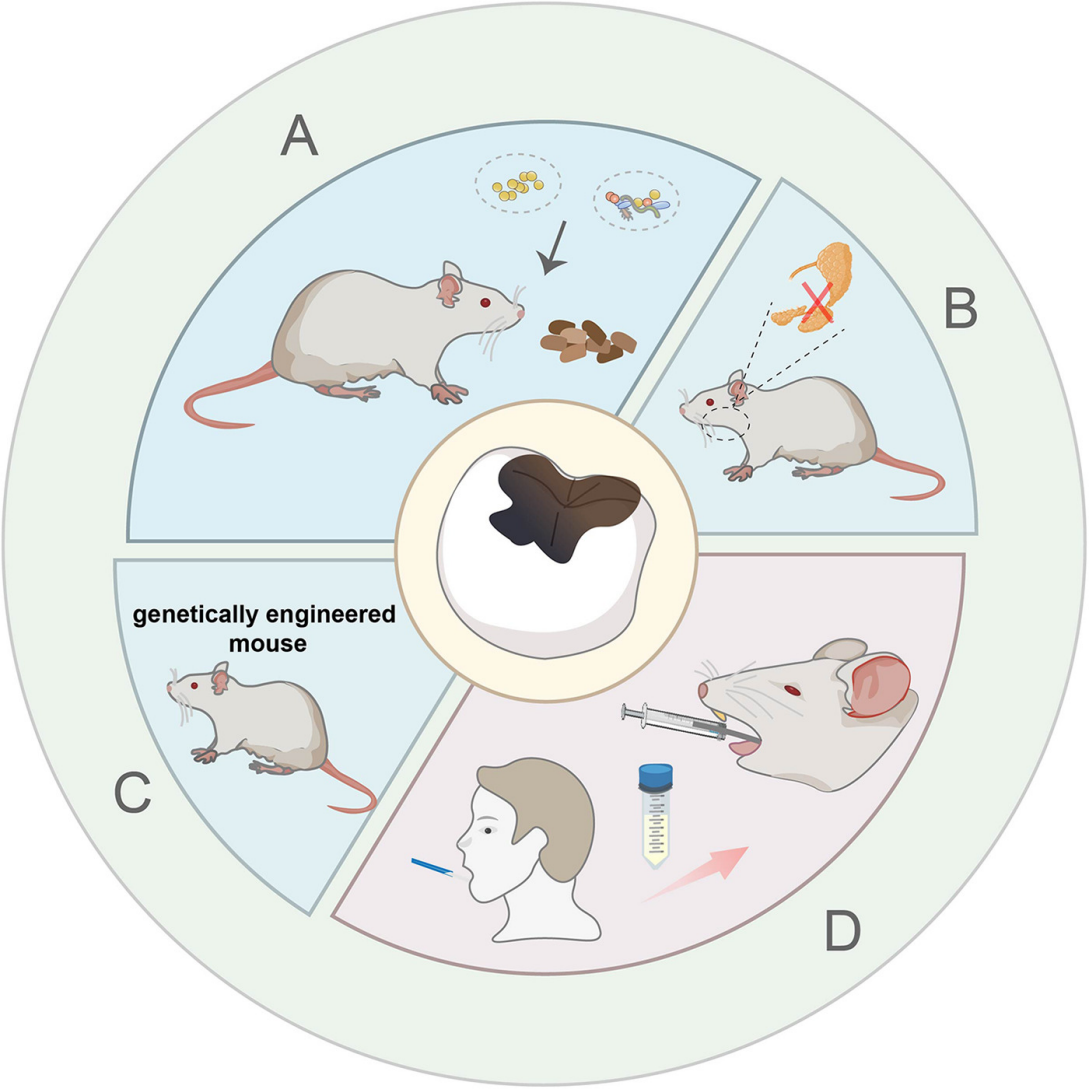
Schematic description of bacterial inoculation induced model with high-sucrose diet. **(A)** Single strains and multi strains bacterial inoculation model. **(B)** Salivary gland resection surgery combined with microbionation model. **(C)** Genetically engineered mouse combined with microbionation model. **(D)** Human oral microbiota-associated mouse model.

Caries is a dynamic, microbial biofilm-mediated, and multifactorial disease involving the destruction of the hard dental tissues (12). It begins with a bacterial infection, followed by biofilm formation and the demineralization of nearby enamel. The formation of caries is closely related to four factors that include oral microorganisms, oral environment, host, and time (13). Hence, frequent intake of carbohydrate can disrupt the ecology of animals' oral bacterial community by predominating acidogenic and acid tolerant species, thereby promoting caries development. In 1958, Keyes proved that dental caries in the molar teeth of rats can be induced by high-carbohydrate low-fat diets (14). Wood et al. fed Sprague-Dawley (SD) rats with 40% sucrose diet for 42 days, forming enamel decalcification and enamel caries on the buccal, proximal, and sulcular surfaces of the rats' maxillary and mandibular molars (15). Instead, BALB/c mice received 10% sucrose containing distilled water and cariogenic diet KEYES #2000 *ad libitum* for 7 weeks showed no demineralization (16). The simple diet induced method has an unsatisfactory modeling effect, and a long-term high sucrose diet can damage animal metabolism, causing obesity, diabetes, etc. Considering the primary pathogen for dental caries was *S. mutans* (17), researchers added it exogenously to the oral cavities of animals to construct dental caries (Figure 1A). They inoculated the rats' teeth with *S. mutans* UA159 for three consecutive days and provided them with Diet 2,000 and sucrose water, successfully constructing caries which damaged the deep layer of dentin (18, 19) (Supplementary Figure S1A). This method is widely adopted by most people since it can shorten the time and establish an unambiguous lesion. Da Silva et al. inoculated rat molars with *S. sobrinus* 6,715 culture for 5 consecutive days. Then, the rats were fed the 2,000 diet and sucrose water for fifty days to induce caries without causing irreversible pulpitis (20). This caries slow progression model facilitates the investigation of the pulp response in carious teeth under different materials.

In addition to bacterial inoculation combined with a cariogenic diet, other measures have been taken to expedite the establishment of caries and simulate the human condition as closely as possible. Some researchers surgically removed the rats' sublingual and submandibular salivary glands and ligated rats' parotid ducts to reduce saliva secretion and created a condition that is more prone to causing tooth decay (21) (Figure 1B). This method requires researchers to have strong technical skills to minimize animal suffering during the operation. Furthermore, Matsumoto N et al. have confirmed that compared with wild mice, the colonization of oral streptococci significantly increased in E2f1-deficient mice which have dry mouth and hyposalivation. He laid a foundation for the genetically engineered mouse to become a useful animal model for dental caries (22) (Figure 1C). Although the inoculation of monospecies bacteria greatly accelerates the formation of caries, the plaque in dental caries is complex and diverse. Pioneer species, such as Streptococc*us sanguinis (S. sanguinis)*, adhere to the tooth surfaces firstly, acidogenic species, such as *S. mutans* and *S. sobrinus* aggregate subsequently, various bacteria continue to grow and develop, and eventually form dental plaque (23). Based on the elevated pathogenicity with combinations of organisms, multispecies bacterial suspension (*S. mutans, S. sobrinus and S. sanguinis*) was put onto the rats' teeth for three consecutive days. Then the rats were fed with a cariogenic diet 2,000 and 5% sucrose water, which caused lots of pit-and-fissure caries after one month (24) (Supplementary Figure S1B). Besides, Wu et al. developed a human oral microbiota-associated mouse model, via gavaging patients' saliva into mice and fed mice with a normal diet for 35 days (25) (Figure 1D). They successfully demonstrated that recipient mice could not only present the oral microbiota of the donors but also display the difference of oral microbiota between the donors by using 16S rRNA sequencing analysis (Supplementary Figure S1C). However, the gavage may cause pain and discomfort to the mice, and needs further improvement.

## Pulpitis

**Figure 2:**
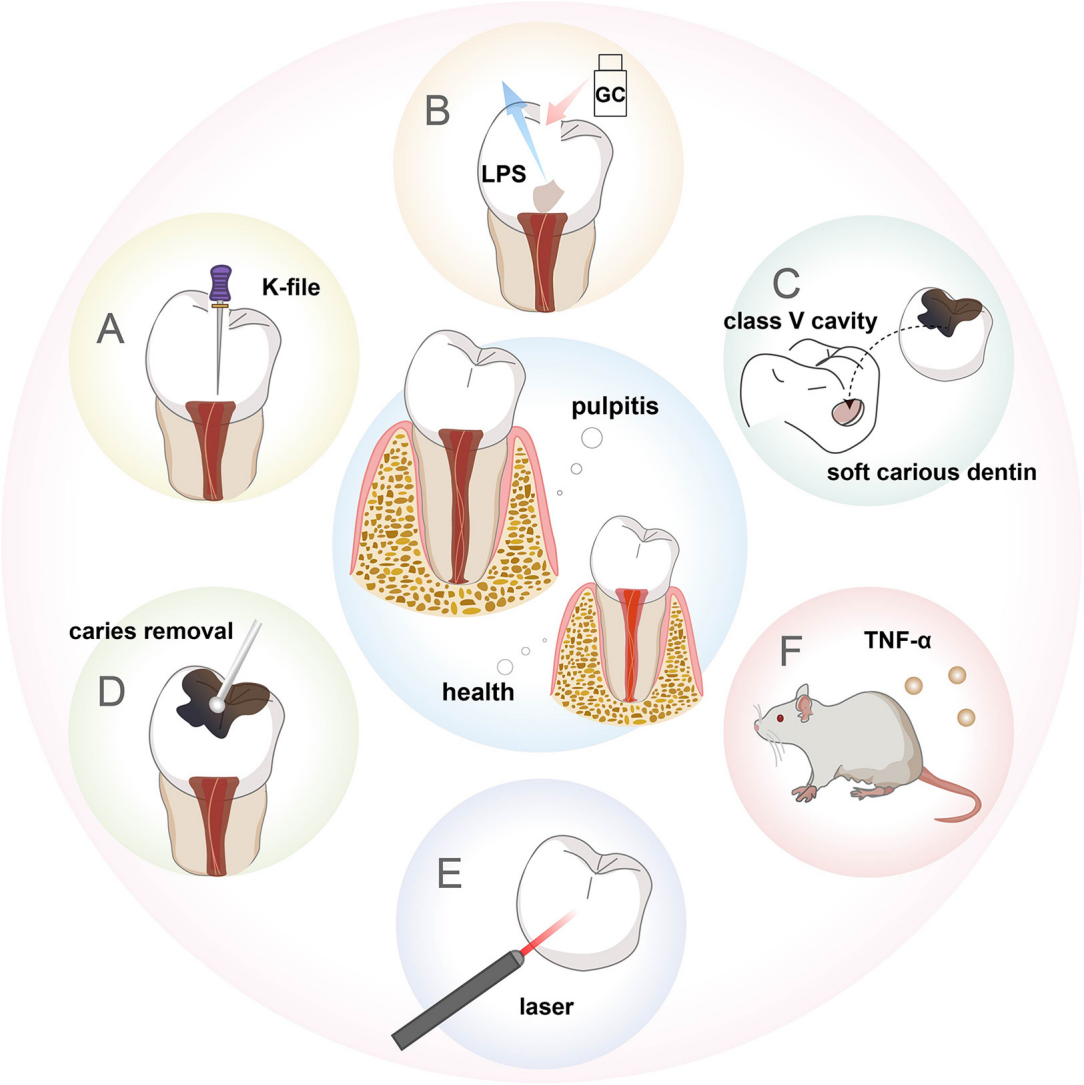
Schematic description of the animal models of pulpitis. **(A)** Pulp exposure model. **(B)** LPS-stimulated pulpitis model. **(C)** Human-derived pulpitis model. **(D)** Caries-induced pulpitis model. **(E)** Laser-induced pulpitis model. **(F)** Transgenic mouse model.

Pulpitis is an inflammatory disease of the dental pulp stimulated by physical, chemical or biological factors, which usually causes pain. Bacterial infection is considered to be the most important trigger of pulpitis (26). *in vivo* pulpitis models are helpful for us to capture the dynamic cellular responses in fully vascularized and innervated pulp tissues. In research, Wistar rats, Sprague Dawley rats and C57BL/6 mice are commonly employed to establish pulpitis models. Pigs (27) and rabbits (28) are sometimes included in the studies as well. Meanwhile some studies explored the potential use of other mouse species, such as BALB/c mice (29) and NMRI mice (30). Since the dental pulp is rich in nerve endings, acute pulpitis can cause severe pain. Therefore, adequate analgesic measures like local anesthetics and painkillers should be applied in the experiment. It is also necessary to closely monitor the changes in the animals' body weight and behavioral activities. The investigator usually uses a round bur and K-file to expose pulp, and leave the exposed preparation cavity open for 24 h (31). Obvious inflammatory cell infiltration can be observed after pulpal exposure (Supplementary Figure S2A). This method rarely leads to early pulp necrosis and requires less time for the actual operation, which may reduce the synergistic effects from a series of irritating stimuli, and facilitate experiments involving a large sample size (Figure 2A). Considering the progress of pulpitis induced by a round bur is relatively rapid, some researchers use a polishing bur to induce a slow progress of pulpal inflammation (32). Necrosis was observed near the exposure site in the polishing bur group at 24 h, while in the round bur group it only took 8 h. However, there is a risk of exposure to unknown sources of inflammation by pulp exposure, which can lead to ambiguous lesions. In this scenario, some add LPS from *Escherichia coli* to the exposed pulp for stable and similar inflammation. The exposed dental pulp was treated with sterile cotton balls soaked in *Escherichia coli* LPS for 15 min, the coronal cavity was then sealed using glass-ionomer cement (GC) for three days (33) (Figure 2B). Diffuse infiltration by numerous inflammatory cells can be displayed (Supplementary Figure S2B). It can stimulate the sudden inflammation induced by oral bacteria in the human pulp exposed after trauma very well, but it cannot represent the long-term and gradual development of the caries process.

Some people prepared class V cavities on the buccal dental surface of dogs, inserted soft carious dentin from freshly extracted human teeth into the floor of the cavity, and filled with glass ionomer (34). This method successfully constructed a model of moderate pulpitis after 7 days and a model of severe pulpitis after 14 days (Figure 2C). However, these studies are based on healthy teeth to investigate effects on hard tissue formation and inflammation. It could not accurately represent the clinical pulpal pathology during caries progression, in which caries-induced inflammation already occurs before pulp exposure. Therefore, Huang et al. built a pulpitis model based on the conventional caries model (Figure 2D). Pulp exposure was intentionally induced with a round bur after complete caries removal in the moderate caries group to construct reversible pulpitis. Pulp was exposed during caries removal in the severe caries group to construct irreversible pulpitis (35) (Supplementary Figure S2C). To explore persistent endodontic infection, Hasan et al. applied zymosan after pulp exposure, thereby producing stable inflammation that could be observed up to 72 h (30). This drugs-stimulated pulpitis model can also be used to study fungal antigen-associated infections of the pulp. In addition to these methods, some researchers chose diode lasers with a wavelength of 970 ± 15 nm (continuous wave, frequency 50 Hz for 60 s) (Figure 2E), controlling the laser output power (1.5, 2.5, or 4 W) to cause varying degrees of pulp damage and repair within 7 days, thus establishing a controlled and quantifiable pulpitis model, which is convenient to study the mechanism of chronic pulpitis (27). Some generated a transgenic mouse model which conditionally overexpressed TNF-α, and bred these mice with a dentin matrix protein 1 (DMP1)-Cre line for overexpression of TNF-α solely in the tooth pulp and bone, forming pulp inflammation resembling pulpitis (36) (Figure 2F).

## Periodontitis

**Figure 3:**
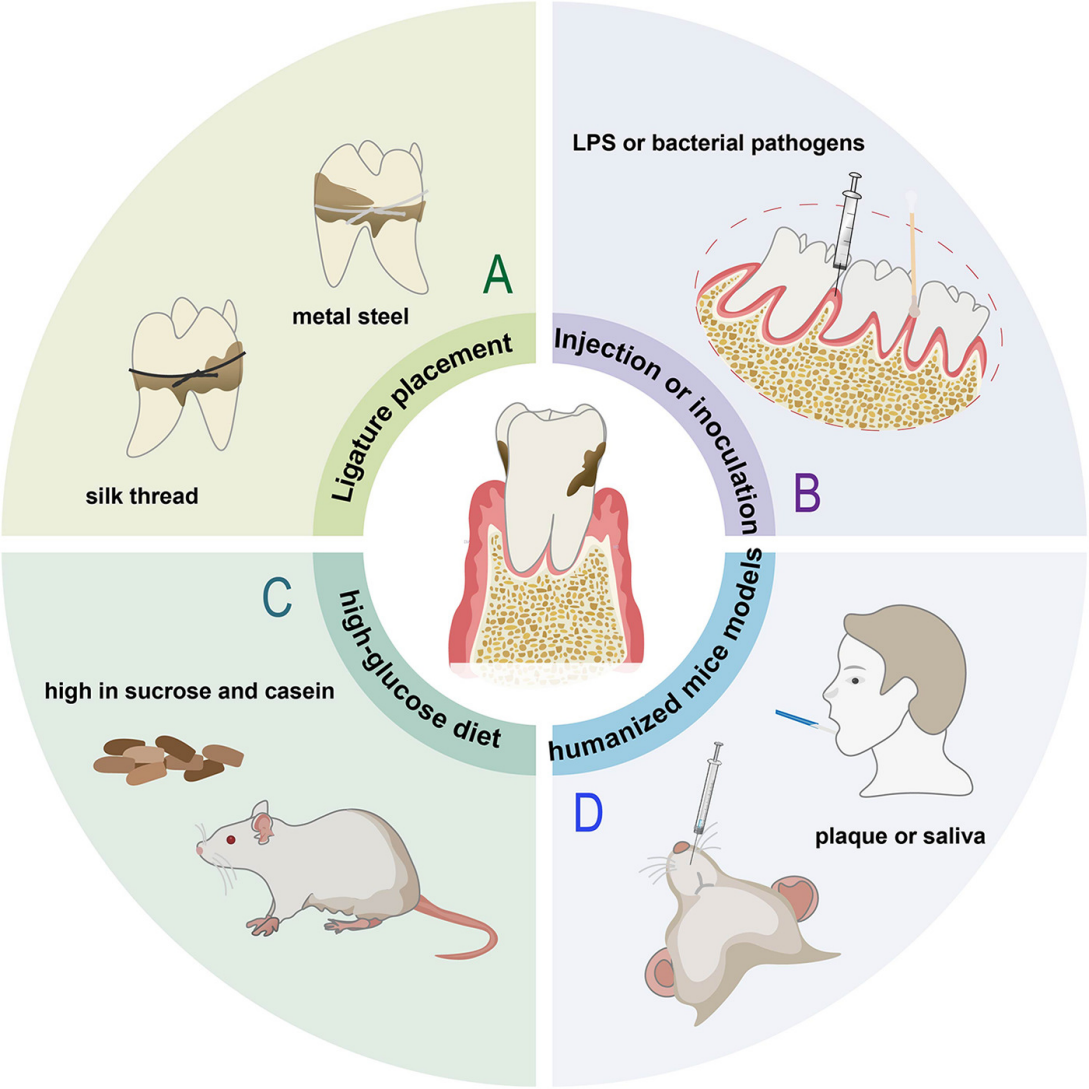
Animal models of periodontitis. **(A)** Ligature placement induced model. **(B)** Rats with injection LPS or inoculation with bacterial pathogens, such as *Porphyromonas gingivalis* (*P. gingivalis*). **(C)** Rats fed a diet high in sucrose and casein. **(D)** Humanized mice injected with subgingival plaque samples or saliva from periodontal patients.

Periodontitis is a multifactorial and polymicrobial disease resulting in periodontal pocket formation, clinical attachment loss, bone defect, and subsequent leading to loss of tooth structure. Non-human primates (37), dogs (38), goats (39) and other large animals can spontaneously develop periodontitis. However, the high cost and special requirements for their care limit their application in periodontal research. The dysbiosis of periodontal microbiota depends on the specific gene combinations or collective virulence activity within the altered microbial community, not so much on the particular microbial roster. Besides, existing studies show the same inflammatory mediators mediate inflammatory bone loss in various species including mice, rats, dogs, non-human primates, and humans. So, rodent models can also be used to study the pathogenesis of human periodontitis (40). Considering rodents have a natural resistance to periodontitis, they need to take some measures to induce periodontitis (41). Because of the advantages such as low price, easy manipulation and availability, ligature only placement is the most common method for periodontitis induction (72.2%) (42), for 2–3 weeks, by using silk thread ligatures (43, 44), metal steel ligatures (45), or a mix of them (Figure 3A), which combines higher robust strength with excellent bacterial aggregation ability (46). For example, Imagawa et al. tied 6-0 silk thread around the left maxillary second molar of Male WT and Spock1-Tg mice to induce periodontitis for 10 days (47), exhibiting obvious periodontal tissue destruction and bone resorption. Among them Spock1-Tg mice exhibited more ligature-induced alveolar bone loss (Supplementary Figure S3A). Ligature induced model promotes biofilm accumulation on the ligatures and stimulation of immune response, resulting in the periodontal destruction (48), but sometimes it could produce mechanical lesions which disturb experimental results. In addition, this method appears to recapitulate acute periodontal inflammation, and is not suitable to reflect the chronicity and persistent dysbiotic state observed in periodontitis of humans (43).

Periodontitis can also be achieved by repeated injection of LPS or inoculation of bacterial pathogens (Figure 3B), most often using *P. gingivalis*, which is considered to be the major etiological factor amongst the pathogenic bacteria involved in the development of periodontitis (48). Considering *Fusobacterium nucleatum (F. nucleatum)* and *Streptococcus gordonii (S. gordonii)* are also important stimuli of inflammation, Wang et al. injected lipopolysaccharide and mixed bacteria (*S. gordonii, P. gingivalis and F. nucleatum*, 1:1:1) into the subgingival area of the mandibular incisor for 3 days (49). The bacterial inoculation model makes pathogenic bacteria colonize the periodontium, thereby instigating an obvious inflammatory response and bone loss (Supplementary Figure S3B), but has the disadvantage with regard to the long time required for significant alveolar bone loss (ABL) to occur (42). No single animal model can reproduce the complexity of periodontitis. Local stimulation alone may not fully reflect natural periodontal disease development, thus Wu et al. injection bacterial and placing ligature wire on rats for 1 week for faster and more definitive progression (50), leading to dark red gingival tissue and white mucosal abscess spots (Supplementary Figure S3C). Unlike the other studies, Kim et al. only ligated mice for one week to form gingival pockets, then removed the ligature and applied *P. gingivalis* for 5 weeks, after that the mice were left for 4 or 8 weeks in cages (51), faithfully mimicking the clinical settings in which perio-pathogens, including *P. gingivalis* colonize around a tooth (Supplementary Figure S3D). Compared to the model ligated alone, the rat periodontitis model by alveolar bone defect combined with silk ligation for 9–12 days manifests a better clinical similarity, significantly higher intensity and a more standardized bone absorption area of experimental periodontitis during the same period (52).

There are also some models of periodontitis induced by special methods, such as, Lewis rats fed a diet high in sucrose and casein for 6 months can develop mild to moderate generalized periodontitis (53) (Figure 3C), C57BL/6 mice injected with Pam2CSK4, a synthetic molecule that mimics bacterial lipoprotein, into the palatal mucosa for 24 days can induce gingival inflammation and alveolar bone loss effectively and reproducibly (54). In order to represent the complexity of human host response to microbial challenge and treatment fully and accurately, non-human primate models or humanized mice models are necessary. Non-human primates can provide a microbiomic analogy to humans, but their cost is extremely high. Regarding humanized mice models, Jiao et al. ligated the left and right maxillary second molar with silk thread and placed in the gingival sulcus. Meanwhile, periodontal pathogens scraped from periodontitis patients were cultured and inoculated. After eight weeks, all the rats were sacrificed (55). The expression of RANKL increases significantly in the periodontitis group (Supplementary Figure S3E). He et al. transplanted saliva of periodontitis patients into the mouse mouth, inducing an immune response (56) (Figure 3D). Periodontitis can also successfully be constructed by bilateral silk ligatures combined with inoculation and engraftment of peripheral blood mononuclear cells from periodontitis patients via intravenous or intraperitoneal (57).

## Oral candidiasis

**Figure 4:**
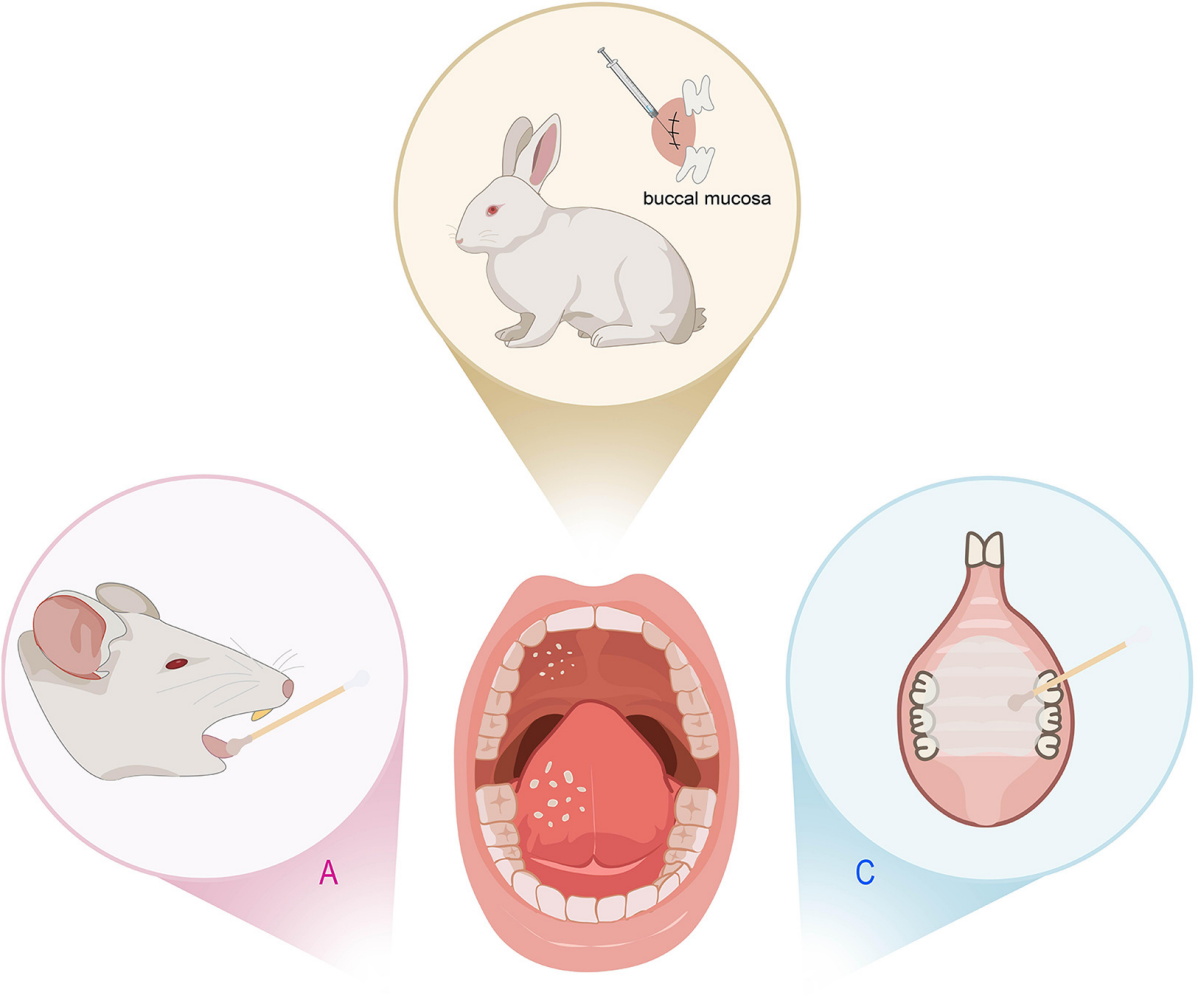
Animal models of oral candidiasis. **(A)** Rats inoculated with *Candida albicans* (*C. albicans*). **(B)** Rabbits’ buccal mucosa scarification injected with yeast solution. **(C)** Rats with a palatal device contaminated with *C. albicans* inoculum.

Oral candidiasis is an opportunistic fungal infection that commonly affects the oral mucosa (58). It is classically seen in patients with dentures, xerostomia, antibiotic and immunocompromise. *C. albicans* is the principal aetiological agent of oral candidiasis. The rodent model, such as rats, mice and rabbits, which are easy to handle and have a low maintenance cost, can reproduce the human candida disease processes and host immune responses very well. However, the above animals are not naturally colonized by *C. albicans*, and candidiasis is prone to develop in the immune-suppressed physiological status due to disease state (59, 60). Thus, in order to mimic the clinical status, some studies have used immunosuppression or antibacterial drugs therapy, reducing the animals' immune response to infection as well as favoring *Candida* colonization. Chen et al. injected mice with cortisone acetate and subsequently placed *C. albicans* cotton swab in the mouth for 2 days (61) (Figure 4A). The epithelium of the tongue is not pronounced or dense, making it more susceptible to fungal colonization. Anwar, S. K. et al. immunosuppressed rats with prednisolone and disturbed the normal oral bacterial flora with tetracycline hydrochloride, then swabbed *C. albicans* cotton pads on the tongues of mice for 1 min (62). Half of the animals had small white lesions in less than 20% of the tongue (score 1), and the other half had large red denuded areas with white pseudomembranes occupying 75% of the tongue's back (score 2) after 4 days from induction of infection (Supplementary Figure S4A). Rabbits are suitable models to simulate buccal delivery, Gajdosova et al. used needles to perform rabbits' buccal mucosa scarification after inducing immunosuppression, and then injected yeast solution to the scarified right buccal mucosa (63) (Figure 4B). Edematous and hyperplastic changes of the scarified and infected buccal mucosa can be observed.

The above protocols inoculate the *C. albicans* directly into the oral cavity of the animals treated with antibiotics or immunosuppressants, resulting in high-incidence and persistent candidiasis infection. However, these strategies can introduce considerable variability. Considering that reducing salivary flow is an important predisposing factor for oral candidiasis, Pérez et al. developed a homogeneous and reproducible model of oral candidiasis due to *C. albicans* in sialoadenectomized rats (64). 100% of the rats showed evidence of infection (Supplementary Figure S4B). Denture stomatitis (DS) is the most frequent type of oral candidiasis. The use of immunosuppressed mice cannot reproduce either the erythematous characteristic of the palate found clinically in candidiasis, nor the systemic condition of immunocompetent denture users. Thus, some studies fitted acrylic devices on the rats' palate (65) or even the combination of both methods (66) (Figure 4C). They inoculated a suspension of *C. albicans* on the immunocompetent rats' palate and followed by the use of a palatal device contaminated with *C. albicans* inoculum for 4 or 7 days. In contrast, some researchers combined intraoral device with multispecies biofilms of *C. albicans, Candida glabrata (C. glabrata), and Candida tropicalis (C. tropicalis)* to induce DS for 4 weeks, but found that it did not induce DS in immunocompetent rats (67) (Supplementary Figure S4C). In oral candidiasis, immunosuppression leaves animals in a vulnerable state, making them highly susceptible to secondary infections that can spread throughout the body and cause tissue damage. Additionally, the tissue edema and ulcer resulting from *Candida* infection often leads to feeding difficulties. Therefore, it is necessary to take some measures to reduce the suffering of animals, such as closely monitor the reactions of the animals, employ local immunosuppressants instead of systemic inhibitors, use non-invasive techniques to monitor infection so that the number of animals sacrificed can be reduced.

## Summary and outlook

We briefly present the characters of oral infectious diseases' animal models, including caries, pulpitis, periodontitis, and oral Candida infection. Because they are also oral infectious diseases, their modeling approach is relatively similar. Oral infectious diseases spontaneously formed by animals usually occur in old age and the lesions are asymmetrical. In order to study the disease mechanism, it is necessary to artificially induce animal disease models to control variables, accelerate the disease progression and reduce the within-group differences. The construction of disease model with pure diet can accelerate this spontaneous phenomenon. But compared with other methods, it still takes a long time and the effect is not clear, making the animals in an uncomfortable state for a long time. The single dominant bacteria induction method takes a short time and can present the pathological state of the disease. However, due to the complex etiology of oral infectious diseases, the model constructed by this method is still quite different from the human model, and the clinical transformation effect is poor, while the multi-strain mixed bacteria induction model is helpful to simulate the complex and changeable microbial environment of the oral cavity. In addition, in pulpitis and periodontitis, researchers often adopt mechanical methods, such as file and orthodontic wire, respectively, to build models, which can sometimes cause mechanical damage to animals, requiring researchers to have corresponding operation techniques to reduce animals' pain. Mechanical method is often used together with bacterial inoculation method to speed up the development process of disease and better simulate human disease.

In recent years, the humanized disease model has attracted attention. In order to closely fit with the clinic, the researchers extracted plaque, calculus, saliva or blood from patients and transplanted them into mice. Related studies have proved that it has a good replicate effect, and can even present differences between different donors. Thus, it is necessary to exclude the interference of other confounding factors in patients, such as smoking, aging and high blood sugar. Besides, certain microorganisms in human saliva or plaque may have unknown effects on animal health and may even trigger new diseases, and more research is needed to explore this. In addition, researchers often take some auxiliary measures to simulate the development process of human disease to help the establishment of animal disease models, such as choosing salivary gland resection in the caries environment, constructing a caries model in pulpitis, inducing pulpitis on this basis, and wearing an acrylic device in the denture stomatitis model to simulate the bacterial infection after long-term wear. The researchers also explored less invasive new methods that greatly reduce the risk of systemic infection and the pain of invasive procedures, such as constructing genetically engineered mice, laser stimulation of pulpitis establishment. Considering the complex etiology of oral infectious diseases, the combination of multiple methods can induce diseases by artificially mimicking multiple etiologies, which may contribute to developing the corresponding animal models. In sum, we hope that by constructing complex microbial environments, we can better simulate the disease conditions occurring in the human oral cavity, providing reference for the study of oral infection.
