# Supplementary material for: Animal models of oral infectious diseases
Source: Front Oral Health. 2025 Apr 17;6:1571492. doi: 10.3389/froh.2025.1571492 (PMC12063496; doi:10.3389/froh.2025.1571492)
Supplement: Supplementary file 1 [file Datasheet1.docx]

Supplementary Material

# Supplementary Figures and Tables

## Supplementary Figures


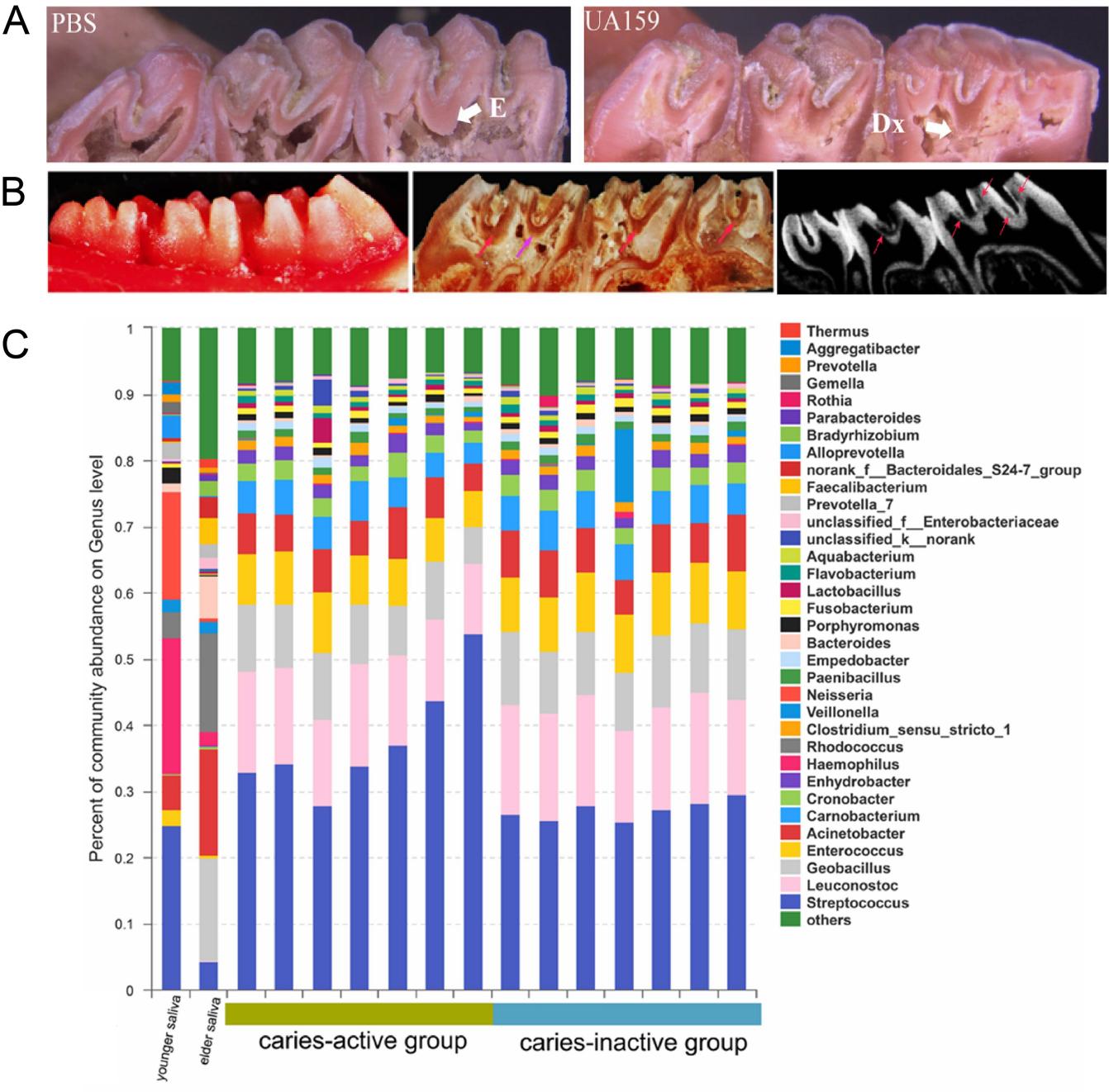


**Figure S1.** Carious lesions assessment of modeling group. **(A)** Pit and fissure caries lesions on the mandibular molars of the uninfected rats and rats inoculated with S. mutans UA159. E indicates that the caries involved only the enamel; Dx indicates that the caries was more than 3/4 of the dentin thickness [18], used under [CC BY](http://creativecommons.org/licenses/by/4.0/). (B) Carious lesions on the smooth surface and sulcal surface with infected teeth. Purple arrows, affected moderate dentinal, 1/4–3/4 of the dentin; red arrows, affected extensive dentinal, beyond 3/4 of the dentin. 2D scale sagittal images of the maxillary molars analyzed by micro-CT. Red arrows, caries lesion sites [24], used under CC BY - NC - ND. (C) Abundance of microorganisms in twins' and mice' saliva [25], used under CC BY.


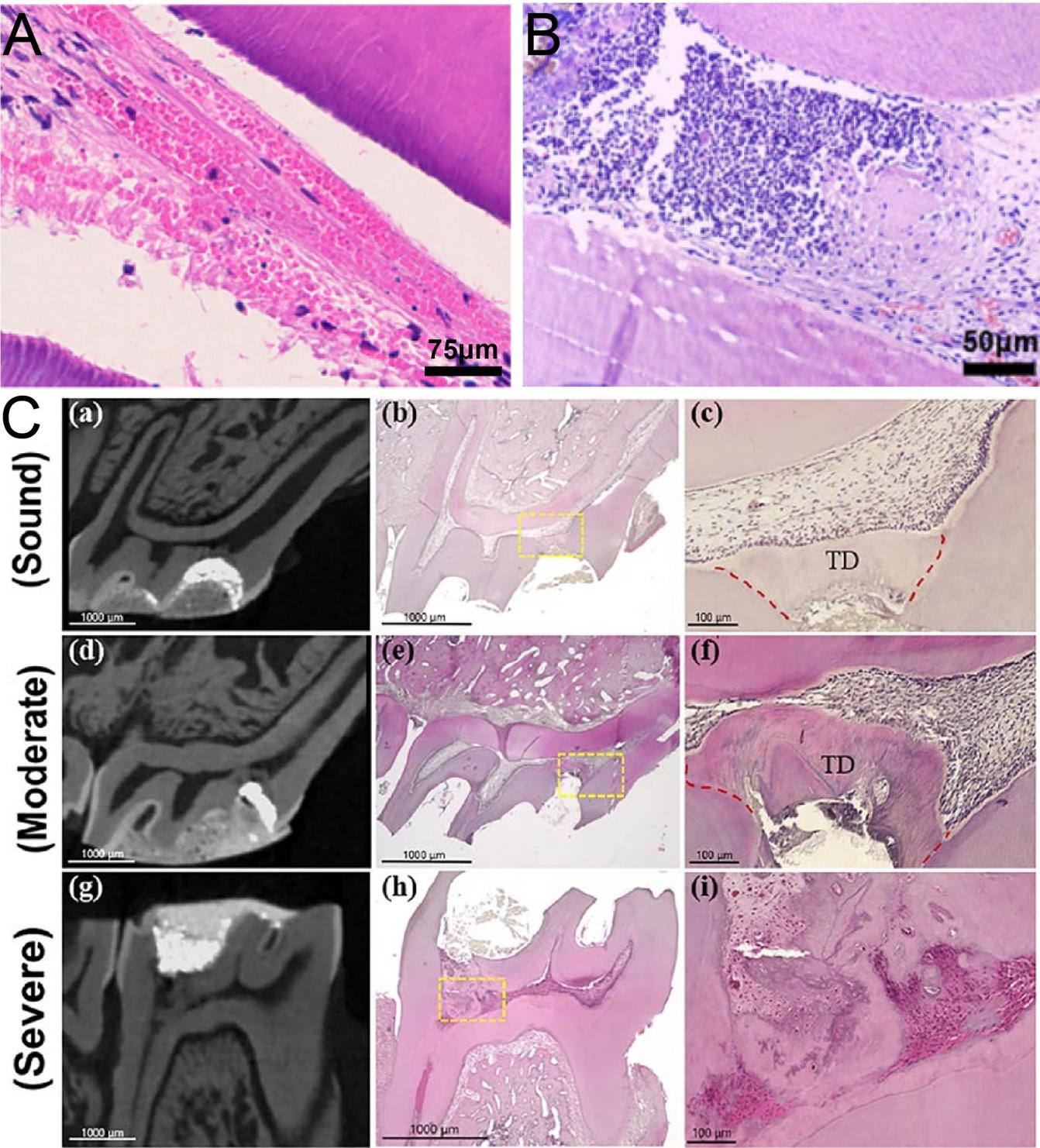


**Figure S2.** Hematoxylin and eosin (HE) staining of dental pulp from modeling group. (A) HE staining of dental pulp from control group and pulpal exposure (PE) group [31], used under CC BY. **(B)** HE staining of LPS-stimulated pulpitis model [33], used under CC BY - NC. **(C)** CT and HE staining images at 4 weeks after pulp capping in the sound (a–c), moderate (d–f), and severe (g–i) groups. Direct pulp capping in sound teeth was used as a control. Red dotted lines indicate borders of newly formed tertiary dentin [35], used under CC BY - NC.


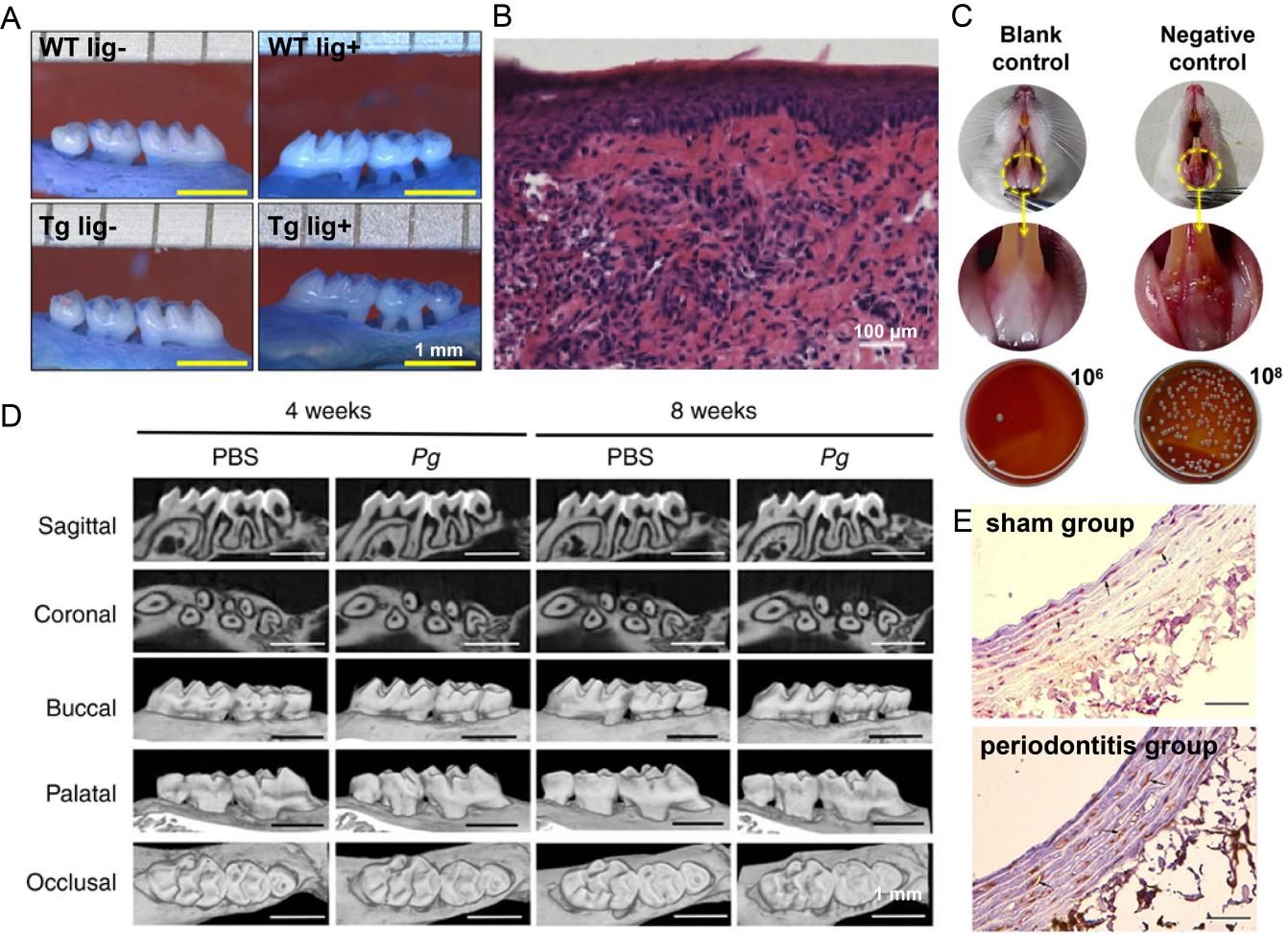


**Figure S3.** Periodontitis lesions assessment of modeling group. (A) The representative photos of alveolar bone loss of the buccal view in WT and Spock1-Tg mice with or without ligature-induced periodontitis [47], used under CC BY. **(B)** HE stained micrographs of periodontal tissues of rats injected with lipopolysaccharide and mixed bacteria [49], used under CC BY - NC -ND. **(C)** Intraoral photos of rats with injecting bacteria and placing ligature wire (negative control) and healthy rats (blank control) [50], used under CC BY. **(D)** Representative two or three dimensional μCT images of mice maxillae. The mice were sacrificed at 4 (right) or 8 (left) weeks after the final PBS or Pg inoculation [51], used under CC BY - NC -ND. (E) Representative vascular immunohistochemistry (RANKL expression) [55], used under CC BY - NC -ND.


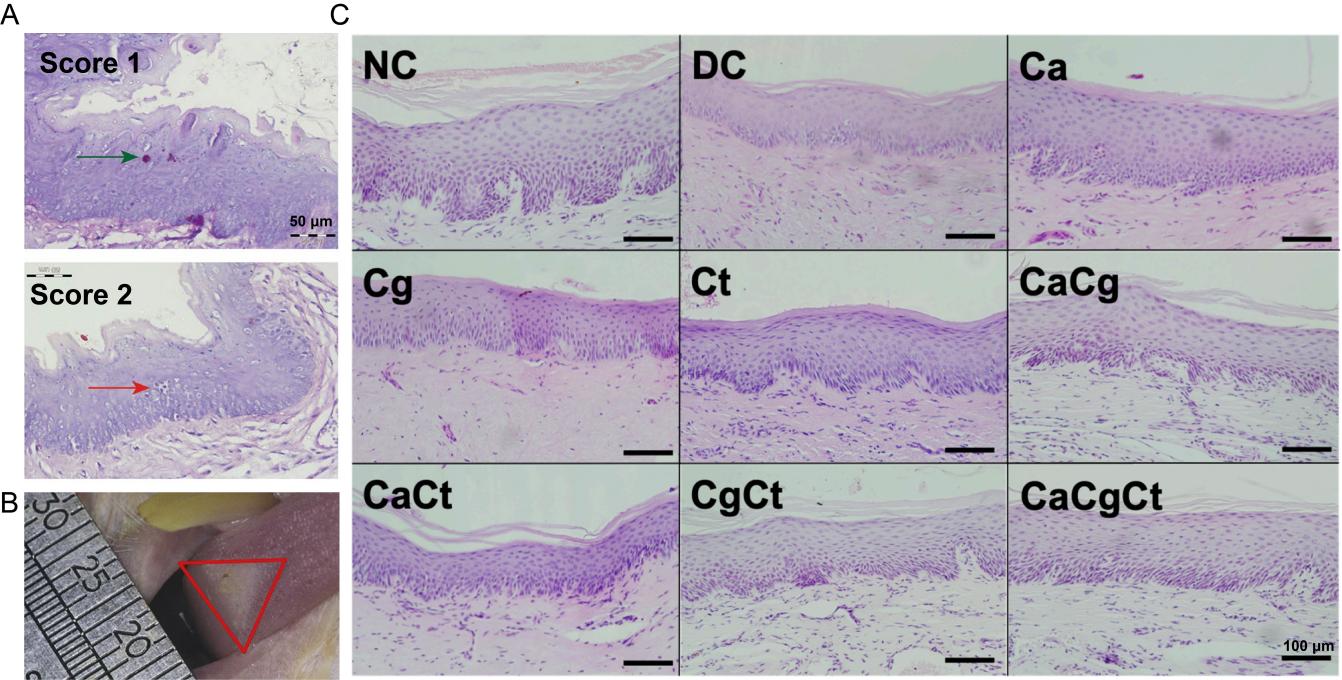


**Figure S4.** Oral candidiasis lesions assessment of modeling group. **(A**) Photomicrographs of PAS-stained tongue specimens at the end of treatment period. The untreated negative controlled tongue mucosa range in their scores from mild to severe [62], used under CC BY. **(B)** Digitalization technique used to evaluate clinical lesions of modeling group [64], used under CC BY – NC - ND. **(C)** Histopathological sections of the palatal mucosa of male rats after the 4 - week period with the acrylic device. NC: negative control. DC: device control, Ca: *C. albicans*, Cg: *C. glabrata*, Ct: *C. tropicalis*, CaCg: *C. albicans* + *C. glabrata*, CgCt: *C. glabrata + C. tropicalis*, CaCt: *C. albicans + C. tropicalis*, and CaCgCt: *C. albicans + C. glabrata + C. tropicalis* [67], used under CC BY.

## Supplementary Tables

|  | **Model type** | **Pathological condition** | **Animal** | **Advantages** | **Disadvantages** | **Refs** |
| --- | --- | --- | --- | --- | --- | --- |
| caries | high-sucrose diet induced model | 40% sucrose diet for 42 days | SD rats | It ideally represents more natural disease states with dysbiosed microbiota by utilizing sucrose and intraoral intrinsic flora. | It takes a long time and the modeling effect is unclear. | [15] |
|  | Bacterial inoculation induced model | *S*. *mutans* for 3 consecutive days and a sugar-rich diet for 5 weeks. | SD rats | This method requires a short time, possessing strong cariogenic properties and good reproducibility. | It can not fully simulate the complex oral microbial environment. | [68] |
|  |  | Multispecies bacterial suspension for 3 consecutive days with a cariogenic diet 2000. | SD rats | It simulates the complex microbial community in the human oral cavity. | It is still different from the caries occurring in humans. | [69] |
|  |  | Sublingual and submandibular salivary glands Resection and parotid ducts ligation with Streptococcus mutans inoculation and a cariogenic diet | Wistar rats | It can simulate the pathological state of reduced saliva secretion very well. | It is invasive, risky and needs complicated operation. | [21] |
|  |  | E2f1-deficient mice with oral streptococci inoculation and 1% sucrose drinking water | E2f1-deficient mice | It showed initial adherence activities of oral streptococci on the tooth surfaces in a manner similar to humans with dry mouth without continuous ingestion of food containing such excess amounts of sucrose. | It is expensive and its stability is unclear. | [22] |
|  |  | Donators' saliva was gavaged into germ-free recipients with normal diet for 5 weeks. | C57BL/6 J mice | It presents the oral microbiota of the donors, perfectly replicating the human caries condition. | It takes a long time. | [23] |
| pulpitis | pulp exposure model | Exposing pulp with 1/4 round bur and #35K-file, then sterile collagen sponge impregnated with PBS for 2 weeks | Wistar rats | It rarely causes pulp necrosis and can collect large samples with short induction time and sample operation. | It could induce a rapid inflammatory process. | [70] |
|  |  | Exposing pulp with polishing bur. | C57BL/6 mice | It induces a relatively slow inflammatory process. | It easily results in perforation. | [32] |
|  | LPS-stimulated model | Exposing pulp by a round bur or #40K-file, and inducing pulpitis by LPS from *Escherichia coli* for 6h. | SD rats | It is less invasive and can induce a stable inflammatory response in a time- and dose-dependent manner in dental pulp cells. | It could induce a rapid inflammatory process. | [71-72] |
|  | Caries-Induced Model | Using a round bur after completing caries removal or during caries removal. | SD rats | It constructs reversible and irreversible pulpitis, accurately representing the clinical pulpal pathology during caries progression, in which caries-induced inflammation already occurs before pulp exposure. | It takes a long time and money to model pulpitis. | [35] |
|  | Others | Developing a transgenic mouse model which conditionally overexpresses TNF-α. | DMP1-Cre mouse | It can mimic pulpitis without extra processing steps. | Other diseases may exist that would interfere with pulpitis. | [36] |
|  |  | Using diode lasers with different output power. | miniature pigs | It can construct reversible pulpitis and irreversible pulpitis. | It may cause damage to other tissues. | [27] |
| periodontitis | Ligature placement induced model | Placing silk ligatures for a month. | Wistar rats | It has advantages such as low price, easy manipulation, availability, similar anatomic structure, and response to periodontal treatment. | It sometimes caused mechanical trauma or ligatures to drop off. | [73] |
|  |  | Placing nylon thread for 20 days. | Wistar rats |  |  | [74] |
|  |  | Removing alveolar bone and tying the teeth with silk ligature. | Wistar rats | It manifests a better clinical similarity, significantly higher intensity and a more standardized bone absorption area. | It requires complex operations. | [52] |
|  | Inoculation with LPS or bacterial pathogens model | *P. gingivalis* oral gavage for 6 weeks. | C57BL6 mice | It causes significant inflammation. | It does not account for the effects of other bacterial pathogens and additional tissue damage would be caused by repeated injections. | [75] |
|  |  | Injecting mixed bacterial suspension (*P. gingivalis:* *F. nucleatum* 1:1) and placing ligature wire for 1 week. | Wistar rats | It constructs a model faster, mimicking the overall host-specific pathogen interactions in human periodontitis. | It is different from human periodontitis. | [50] |
|  | High sucrose and casein diet model | a diet high in sucrose and casein for 6 months. | Lewis rats | It develops mild-to-moderate generalized periodontitis without performing additional mechanical oral intervention. | The high sugar factor interferes with the experimental results. | [53] |
|  | Humanized mice | Injecting pooled subgingival plaque (SP) samples for 21 days with normal food and water. | SD rats | It induces a human like periodontitis condition in which the composition of mouse periodontal bacteria matched better with that of periodontitis patients. | Clinical patient recruitment is required. | [76] |
|  |  | 5/0 silk suture and SP samples application for 14 days. | C57/B6J mice |  |  | [77] |
| Oral candidiasis | Inoculating bacterial model | Immunosuppressing with prednisolone and inoculating with C. albicans for 5 days. | BALB/c mice | It provides a simple and reproducible oral candidiasis model. | It introduces considerable variability. | [78] |
|  |  | C. albicans after Sialoadenectomy on 2 consecutive days. | SD rats | It develops a homogeneous and reproducible model of oral candidiasis without immunosuppressor. | There are certain technical requirements for researchers to perform sialoadenectomy. | [64] |
|  | mucosa scarification with bacterial injection model | Injecting *C. albicans* into buccal mucosa scarification and immunosuppressing with dexamethasone sodium phosphate, enhancing the infection with benzylpenicillin for 3 days. | European rabbits | It establishes a suitable model to simulate buccal delivery associated with candidiasis. | There are individual differences among rabbits with buccal mucosa scarification. | [63] |
|  | palatal device placement | Palatal device contaminated with *C. albicans* and  cemented with a self-adhesive resin cement for 4 days. | Wistar rats | It infects the palate and tongue simultaneously and presents the systemic condition of immunocompetent denture users. | Many manufacturing steps are required to create the device. | [66] |

68. Jiang W, Wang Y, Luo J, Chen X, Zeng Y, Li X, et al. Antimicrobial Peptide GH12 Prevents Dental Caries by Regulating Dental Plaque Microbiota. *Appl Environ Microbiol*. (2020) 86(14). doi: 10.1128/AEM.00527-20

69. Zhang Z, Ji Y, Liu D, Zhou S, Wang Z, Chen R, et al. Heat Shock Protein Inhibitors Show Synergistic Antibacterial Effects with Photodynamic Therapy on Caries-Related Streptococci In Vitro and In Vivo. *mSphere*. (2023) 8(2). doi: 10.1128/msphere.00679-22

70. Cao RY, Wang Q, Wu JM, Liu MM, Han Q, Wang XY. Nell-1 attenuates lipopolysaccharide-induced inflammation in human dental pulp cells. *J Mol Histol*. (2021) 52(4):671-80. doi: 10.1007/s10735-021-09976-y

71. Minic S, Florimond M, Sadoine J, Valot-Salengro A, Chaussain C, Renard E, et al. Evaluation of Pulp Repair after Biodentine<SUP>TM</SUP> Full Pulpotomy in a Rat Molar Model of Pulpitis. *Biomedicines*. (2021) 9(7). doi: [10.3390/biomedicines9070784](https://doi.org/10.3390/biomedicines9070784)

72. Li M, Tian J, Xu Z, Zeng Q, Chen W, Lei S, et al. Histology-based profile of inflammatory mediators in experimentally induced pulpitis in a rat model: screening for possible biomarkers. *Int Endod J*. (2021) 54(8):1328-41. doi: 10.1111/iej.13514

73. Dogan SSA, Toker H, Goze oF. Melatonin Decreases Alveolar Bone Loss in Rats with Experimental Periodontitis and Osteoporosis: A Morphometric and Histopathologic Study. *Biomedicines*. (2024) 12(3). doi: [10.3390/biomedicines12030684](https://doi.org/10.3390/biomedicines12030684)

74. Ferreira-Fernandes H, Barros MAL, Souza MD, Medeiros JVR, Vasconcelos DFP, Silva DA, et al. Topical application of cashew gum or chlorhexidine gel reduces overexpression of proinflammatory genes in experimental periodontitis. *Int J Biol Macromol.* (2019) 128:934-40. doi: 10.1016/j.ijbiomac.2019.02.002

75. Elashiry M, Carroll A, Yuan J, Liu Y, Hamrick M, Cutler CW, et al. Oral Microbially-Induced Small Extracellular Vesicles Cross the Blood-Brain Barrier. *Int J Mol Sci*. (2024) 25(8). doi: 10.3390/ijms25084509

76. Datey A, Thaha CSA, Patil SR, Gopalan J, Chakravortty D. Shockwave Therapy Efficiently Cures Multispecies Chronic Periodontitis in a Humanized Rat Model. *Front Bioeng Biotechnol*. (2019) 7. doi: 10.3389/fbioe.2019.00382

77. Bai L, Chen B-Y, Liu Y, Zhang W-C, Duan S-Z. A Mouse Periodontitis Model With Humanized Oral Bacterial Community. *Front Cell Infect Microbiol*. (2022) 12. doi: 10.3389/fcimb.2022.842845

78. Mima EGdO, Pavarina AC, Jordao CC, Vieira SM, Dovigo LN. Curcuminoid-Mediated Antimicrobial Photodynamic Therapy on a Murine Model of Oral Candidiasis. *J Vis Exp*. (2023) 200. doi: 10.3791/65903
